# Dreaming and Neuroesthetics

**DOI:** 10.3389/fnhum.2015.00348

**Published:** 2015-06-23

**Authors:** Umberto Barcaro, Marco Paoli

**Affiliations:** ^1^Department of Computer Science, University of PisaPisa, Italy; ^2^State LibraryLucca, Italy

**Keywords:** dreaming, Neuroesthetics, Giorgione, Leonardo da Vinci, Vermeer, Millais

## Abstract

This paper, which is limited to the art of painting, aims to support the idea that a substantial insertion of concepts and methods drawn on dream psychology and dream neuroscience can contribute to the advancements of Neuroesthetics. The historical and scientific reasons are discussed that have determined the so far poor role played by the dream phenomenon in the developments of Neuroesthetics. In the light of recent advancements in psychophysiological research, a method of analyzing artistic products is proposed that is based on the recognition of precise features proper of the dreaming experience. Four examples are given for application of this method, regarding works by Giorgione, Leonardo da Vinci, Vermeer, and Millais, respectively.

## Introduction

The aim of this paper is to support the idea that a substantial insertion of contents drawn on dream psychology can contribute to the advancements of Neuroesthetics. Our analyses are limited to the art of painting.

The paper is divided into nine sections. After the present Introduction Section, the second and the third section are focused on the diffused awareness among artists and among dream scholars, respectively, of the role of dreaming experiences in art production. In the fourth part, remarkable results obtained in the field of Neuroesthetics are shortly summarized and the poor role played by the dream phenomenon is underlined. The fifth section is dedicated to the reasons that have determined this underestimation. Then, considering significant aspects of current psychological and physiological research, the discussion involves how these reasons can be overcome. In the seventh section, precise points are indicated that can help introduce an effective analysis of artistic products in the light of phenomenological features proper of dreaming. Among these points are: the establishment of unexpected powerful links between different memory sources; the significance of these links according to recent research on dream psychology; the dream-like character of phenomena of scale invariance; the interplay, in the artist’s creative process and in the observer’s esthetic response, between different levels of consciousness that are connected to dreaming. In the eighth section, four detailed examples are given of application of these concepts to important paintings in the history of art. Finally, a short Conclusions Section summarizes the main points of the proposed approach.

## The Diffused Awareness Among Artists of a Connection Between Art and Dreaming

The artistic representation of dreams has been frequent throughout history; however, the idea of a close connection between art and dreaming has only become almost pervasive in the last century. In fact, a turning point in art history was provided by Surrealism. In the “Manifesto of Surrealism”, which appeared in 1924, Breton defined “Surreality” as “a kind of absolute reality” provided by the solution of the “seeming contradiction” between dream and reality. According to the “Manifesto”, Surrealistic art was based on a direct, “automatic” correspondence with psychological phenomena: by means of “psychic automatism in its pure state”, the Surrealists could “express the actual functioning of thought” (Breton, 1969). An extreme application of the criterion of automatism was performed by Dal*ί*: in a “delightful eccentric volume” (Barrett, 2001) written in 1948, he instructed aspiring artists, partly jokingly and partly seriously, how to find inspiration by being “progressively invaded by a serene afternoon sleep” (Dalί, 1992), actually suggesting that they should draw images from hypnagogic experiences. Of course, the Surrealistic approach to art did not appear suddenly, but was preceded by a number of historically important anticipations: well-known examples are given by the pre-Raphaelites and the Impressionists and, tracing back to a more remote past, by painters who, like Bosch, were distant from a realistic depiction of nature. After the “Manifesto”, the ideas of Surrealism have remarkably influenced all the subsequent artistic movements and all the approaches to the study of art. Today, most artists are thoroughly aware that a close correspondence exists between art and the inner world of both the artist and the observer, and that dreams can reveal significant contents of this inner world. Indeed, in spite of the increasingly complex aspects of current approaches to art, their historical connection to the ideas proper of Surrealism generally appears as fundamental. A great number of poets, painters, sculptors, musicians, and film-makers have explicitly and diffusedly witnessed the value of dreams for their artistic inspiration.

## Recent Contributions of Dream Scholars to the Study of the Role of Dreams in the Creation of Artistic Products

The awareness of the connection between art and dreaming has been also shared by dream scholars, who, especially in the last decades, have often stressed the importance of dreams in the creation of artistic products.

Each issue of “Dreamtime”, a magazine published by the International Association for the Study of Dreams (IASD), contains a section about dreams and movies and several articles about dreams and visual arts. Each annual conference of the IASD includes the exhibition of artistic works representing dreams: some of these works have been digitally duplicated and made available on the website of the IASD Art Gallery. A special issue of the journal “Dreaming”, which appeared in 2003, has been dedicated to “Dreaming and the arts”, under the perspective of investigating how “the study of dreaming can shed light on the creative process, and vice versa” (Russo, 2003). In this issue, specifically with regard to visual arts, States (2003), focusing on the Surrealists and other typical authors of dream-like paintings (De Chirico, Delvaux, Picasso, and Dal*ί*), expresses the opinion that the appeal of the works of these painters is due to the human innate attraction to distortions of reality. Bogzaran (2003), reflecting on her own experience as a dreamer and artist, indicates two particular modalities of dreaming, i.e., lucid dreaming and hypnagogic dreaming, as powerful sources for artistic inspiration. Still in the same issue, Bulkeley (2003) examines the influence of dreaming on the filmmaking of David Lynch, whose works have often been inspired by his personal dream experience and often represent characters that talk or sing about dreams and even dream of dreams. Bulkeley observes that “Lynch’s filmmaking offers an excellent case study of the powerful connection between dreaming and movies in contemporary American society”. A basic point for Bulkeley’s analysis is that the dreaming experience provides a model for the narrative structure of Lynch’s movies. He makes a comparison between movies like “Mulholland Drive” and “Lost Highway” with “certain Hindu myths in which people become so entangled in each other’s dreams and dreams-within-dreams that readers cannot help but feel confused about the basic existential question of ‘what is real”’.

The first chapter of Barrett’s book on dream creativity (Barrett, 2001) is dedicated to visual arts, “which reproduce natural dream imagery most closely”. In this chapter, a large number of instances are given of a lasting close relationship between visual artistic products and dreaming, from the prehistoric paintings in the caves of Lascaux to contemporary painters, sculptors, and even architects. In addition to Surrealism, the importance of dreams is underlined with regard to great painters that preceded Surrealism, including Dürer, Blake, and the Pre-Raphaelites. As for more recent artists, Barrett highlights how complex the relationship can be between art and dreaming. For instance, Magritte, whose paintings are commonly referred to as examples of dream art, “never used nighttime dreams in his work”; another example is Bulgakoza’s “Dream about the red bird”, painted in 1988 (Kornetchuk and Roberts, 1991), which shares the properties of a “phantasmagoric dream” with those of a “political allegory”.

As appears from the just mentioned literature, dream scholars maintain that a connection between visual art and dreaming is general and greatly significant. They have observed that this connection regards not only the representation of dreams but also a form of structural similarity: this point has been remarked by Bulkeley in the above quoted observation about the narrative of Lynch’s movies as well as, with regard to visual arts, by Barrett, who often refers to “dreamlike, but not dreamed, structure”. This point plays a basic role for the point of view that we are presenting in this paper.

## The Poor Role of Dreaming in the Current Discipline of Neuroesthetics

A first understanding of the psychology of esthetic experience originated together with modern science, when Galilei (1623) distinguished between “primary” and “secondary” properties, especially with regard to music; this distinction was developed in particular by Locke (1689/1690). Further periods of significant progress were due to British empiricism in the mid-18th century, and, in the second half of the 19th century, to the theory of evolution and to early neurosciences. In a historical perspective (see Nadal and Pearce, 2011), great advancements were provided by the foundation, still in the second half of the 19th century, of scientific experimental psychology and empirical esthetics (Fechner, 1860, 1876; Helmholtz, 1863; Wundt, 1873–1874). Indeed, for the first time in the history of science, Fechner’s “Vorschule der Aesthetik” offered an approach to esthetics totally based on an empirically grounded study of perception. For instance, his research about the ideal proportions for a rectangle, far from being derived from abstract assumptions, was performed by collecting psychophysical data from people of different ages and educational backgrounds: in this way the golden rectangle, which had been deemed “a standard, even Ideal, for beauty throughout history”, was “reduced to a mere percentage” (Hetrick, 2011).

The birthdate of the current discipline of Neuroesthetics is often indicatively placed in the last decade of the last century. Of course, this is a simplification, because important studies had earlier resumed the tradition of empirical esthetics (e.g., Berlyne, 1971). In the paper “The neurology of kinetic art” (Zeki and Lamb, 1994), the principle that the fruition of visual art follows the rules of visual perception was clearly stated on the basis of recent developments in neurosciences and was supported by examples taken from artistic products. More precisely, the authors focused on the perception of movement as “an autonomous visual attribute, separately processed [in the visual cortex] and therefore capable of being separately compromised after brain lesions”: they viewed kinetic artists as somehow engaged in “exploring the organization of the visual brain though with techniques unique to them”. In the same year, the article “Art and Neuroscience” (Changeux, 1994), a modified English version of a work published in French in 1988, proposed an approach, derived from Gombrich (1960), that aimed to explore the relationship between visual perception, knowledge and pictorial images “in Darwinian terms”. Considering studies on patients that presented brain lesions (Luria, 1966), Changeux stated that “esthetic pleasure apparently results from the initiation of a resonance as well as from the concerted mobilization of groups of neurons located on several levels of cerebral organization of the brain, from the limbic system to the frontal cortex”. One year later, in the book, written in Italian, “Arte e cervello”, Maffei and Fiorentini (1995) described various forms of connection between art and properties of visual perception. For instance, they showed that the optical illusion of the so-called Mach bands was exploited in a number of artistic works, including paintings by Mantegna, Signac, and Seurat, as well as Korean jars of the 18th Century. Zeki (1999) described how different areas of the brain respond to the various fundamental elements (color, form, line and motion) of visual artistic products. According to Zeki, selectivity, search for essentiality, and removal of superfluous are proper of visual art, which therefore should be viewed as an extension of the major functions of the visual brain. A special number of the “Journal of Consciousness Studies” dedicated to “Art and Brain”, also published in 1999, contained a number of significant contributions, in particular the editorial introduction by Goguen (1999) and a paper by Ramachandran and Hirstein (1999), in which eight “laws of artistic productions” were listed and discussed. Among these laws, which, according to the authors, the artists apply “either consciously or unconsciously”, is the psychological effect called “peak shift”: if the recognition of a perceptual feature of a stimulus is rewarded, the response of the subject is more vigorous if this feature is enhanced. According to Ramachandran and Hirstein, grouping is a fundamental principle as well: “the different extrastriate visual areas may have evolved specifically to extract correlations in different domains (e.g., form, depth, color), and discovering and linking multiple features (“grouping”) into unitary clusters—objects”.

An important, fruitful change in the field of Neuroesthetics took place about one decade ago. In the year 2004, three papers reported innovative applications of neuroimaging to the esthetic appreciation of paintings. Cela-Conde et al. (2004) applied magnetoencephalography to show that the prefrontal area was selectively activated by objects that the observers qualified as “beautiful”. Kawabata and Zeki (2004) applied fMRI and found that the perception of different categories of paintings was associated with distinct and specialized visual areas of the brain. Vartanian and Goel (2004) applied fMRI to subjects that were observing paintings and had been instructed to rate them from the esthetic point of view: a quantitative correlation was thus found between esthetic rating and activation of specific neuroanatomical regions. Two years later, Jacobsen et al. (2006) applied fMRI to subjects who were observing graphic patterns and compared neural activity while participants were rating “beauty” with neural activity while participants were rating “symmetry”: the authors found that the judgment of beauty relied on a network partially overlapping with networks connected to stimulus complexity and symmetry. In recent years, neuroimage studies have further developed: we limit ourselves to mentioning Di Dio et al. (2007), who studied brain responses to Renaissance sculptures, Cela-Conde et al. (2011), who provided a critical review of the information that neuroimaging methods had so far provided about the cognitive and neural underpinnings of esthetic appreciation, and Ishizu and Zeki (2014), who found that the experience of “sublime” and that of “beautiful” engaged distinct brain systems. Furthermore, other basic issues have been studied, including: the effect of neurological syndromes on artistic production (Chatterjee, 2004); evolutionary perspectives (Nadal and Pearce, 2011); specific approaches to different forms of art (Chapters from 8 to 12 of Skov and Vartanian, 2009; Vartanian et al., 2015); the possible role of the mirror neuron system (Friedberg and Gallese, 2007); the role of theories of emotion (Brown and Dissanayake, 2009); the effects of transcranial current stimulation (Cattaneo et al., 2014).

This concise summary of the developments of Neuroesthetics shows that so far the experience of dreaming has played no role, or at least a very little role. In the next section, we discuss the historical and scientific reasons for this fact.

## Reasons for the Underestimation of the Dreaming Experience in Current Neuroesthetics

The psychological approach to esthetics in the 19th century took advantage of the great advancements in the knowledge of perception given by experimental psychology and neurophysiology. The total exclusion of dreaming from the scope of empirical esthetics is historically obvious: in the 19th century the scientific approach to dreaming was scarcely developed, although some historically significant results had already been obtained (for a review, see Chapter 18 of Finger, 1994).

In 1899, Freud’s “Traumdeutung”, although being based on an innovative approach and presenting deep insight into the human mind, was however very far from the requirements for rigorous scientific research. In the following decades, the issue of dreaming continued being important for the various psychoanalytical approaches, but remained substantially extraneous to the methods of properly scientific disciplines, such as behavioral psychology, and later, in the second half of the 20th century, cognitive psychology. Very indicatively and schematically, we can historically place the definite insertion of dreaming into the discipline of physiology in 1953, when Aserinsky and Kleitman (1953) connected the psychological phenomenon of dreaming with the physiological phenomenon of the periodic occurrence of rapid eye movement (REM) sleep during the night. However, in the second half of the century, physiology and psychology of dreaming were generally separate and often conflictual. The identification of dreaming with REM sleep was subjected to criticism; physiological theories of dreaming often only found limited confirmation, psychological models and therapeutic uses of dreaming were extremely different from each other and often contradictory. The results offered by the cognitive approach to the study of dreams (Cavallero and Foulkes, 1993), although being obtained in a rigorously scientific way, did not appear directly applicable to the study of artistic experience. Thus, the basic reason for the exclusion of dreaming from the scope of Neuroesthetics was that the physiological study of the dreaming brain was much less developed than the physiological study of perception. Another important reason was that the automatic and quantitative analysis of natural language and verbal data was only beginning: indeed, the knowledge of dream contents can only be obtained by means of written or oral words. Certainly, scientists active in Neuroesthetics knew the role of dreams in the surrealistic art; however, the connection between art and dreaming appeared more close to psychoanalysis, generally viewed as non-scientific, than to properly scientific approaches.

The great change in Neuroesthetics that occurred about one decade ago, and is still influencing current research, was based on the idea of applying to esthetics the new effective tools of neurosciences, i.e., the increasingly powerful methods of acquiring and processing not only electrophysiological signals, but also three-dimensional images of the neural system. In other words, recent research in Neuroesthetics has been motivated by the awareness that the new techniques offer the possibility of an effective insight into various perception modalities in the particular condition of esthetic experience, which takes place during wakefulness. For these reasons the absence, or very little presence, of the issue of dreaming in the recent developments in Neuroesthetics has contributed to its advancements. At the same time, we feel that just these advancements can pave the way for a fruitful insertion of dreaming into this discipline. In the next section, we discuss why we feel that the insertion can now take place.

## Recent Scientific Developments that Suggest that References to Dreaming can Contribute to the Advancements of Neuroesthetics

The reasons indicated in the previous section are now being overcome. A first reason is that the physiology of REM sleep is better known under many aspects, including changes of cortical activity with respect to wakefulness and NREM sleep (Steriade et al., 2001; Destexhe et al., 2007; Vyazovskiy et al., 2009), changes in the activity of neuromodulators (see, e.g., Monti and Monti, 2007), low-frequency (less than 0.1 Hz) interactions between brain regions (Chow et al., 2013), and properties and functions of a thalamocortical network activated during REM sleep (Wehrle et al., 2007). A basic tool for the achievement of these results has been provided by functional fMRI.

Among the data provided by the physiological investigation of REM sleep, a distinction between tonic and phasic epochs is particularly interesting, because it demonstrates that REM sleep is far from uniform: phasic epochs are characterized by distinct oculomotor activity (see, e.g., Sallinen et al., 1996), increase in arousal threshold (Ermis et al., 2010), reduction in the alpha power of the EEG signal (Cantero et al., 2000), and increase of major motor activity in patients affected by REM sleep behavior disorder (Frauscher et al., 2009). Furthermore, epochs exist that are specifically characterized by slow eye movements (SEMs; see, e.g., Marzano et al., 2007; Pizza et al., 2011), whose frequency range is approximately the same as that of eye movements during Stage 1. The presence of SEMs both during REM sleep, the privileged condition for dreaming, and during Stage 1, the privileged condition for hypnagogic hallucinations, suggests that SEMs could be directly connected with fundamental properties of sleep mentation. In the light of these results, REM sleep can be subdivided into three sub-stages (Magrini et al., 2014): epochs presenting reduced eye movements, epochs presenting REMs, and epochs selectively presenting SEMs.

The study of the very complex relationship between REM sleep and dreaming has recently advanced by means of new experimental data and critical analyses. According to Nir and Tononi (2010), “refined spatial analysis using fMRI or high-density EEG could identify regionally specific predictors of dreaming”. The role of REM in specific consolidation of emotional memory has been investigated (Payne et al., 2012). Attempts have been made to study the neural decoding of visual imagery during sleep: for instance, Horikawa et al. (2013) have found links between brain activation patterns and verbal reports by applying lexical and image databases to the analysis of mentation during sleep onset. The idea that REMs are connected to the observation of dream images has been more clearly defined by means of a “revised scanning hypothesis” (Hong et al., 2009). Indeed, the original scanning hypothesis (see e.g., Herman et al., 1984) concerns a correspondence between the direction of the dreamer’s eye movements and the movements of objects in the dream. The revised scanning hypothesis adds a different kind of movement, not associated with awareness of gaze direction, similar to those that occur in wakefulness during the process of scanning stationary objects.

As underlined by Nir and Tononi (2010), an issue currently faced by research on REM sleep regards the debate between two opposite hypotheses about the generation of dreams. According to the bottom-up view, supported in particular by Hobson’s model (Hobson and McCarley, 1977; see also Hobson et al., 2000), dreams are due to a synthesis, performed by high-order brain areas, of data produced by low-level sensory areas. Differently, according to the top-down view, dreams begin as wishes, abstract thoughts and memories which are enriched with perceptual features.

An important source for the neuropsychology of dreaming has been given by Solms (1997), who examined a large number of neurological patients and observed that dreaming depended on specific forebrain regions. It is interesting to observe that the neuro-anatomical approach of Solms led the author to find possible correspondences with psychoanalytical concepts.

The psychological study of dreaming has recently taken advantage of the great progresses in the automatic and quantitative analysis of language. Certainly, the scientific community is now more aware that verbal data are objective outputs of physiological systems as much as electrophysiological signals are. The application of statistical methods to verbal data has allowed dream content to be analyzed and compared among different conditions by means of the diffused application of the Hall/Van de Castle system, introduced towards the end of the 20th century (Domhoff, 1996; Hall and Van de Castle, 1996; for a more recent critical analysis, see Schredl, 2010). Important aspects of dreaming have been quantitatively studied, including dream recall, presence of emotion in dreams, frequency of lucid dreaming and of nightmares, connection between dream contents and waking experience. Dream questionnaires have been implemented to elicit information about these issues and about attitudes towards dreams (see, e.g., Schredl et al., 2014). Analysis of verbal data including associations has allowed researchers to better understand the possible significance of single dreams (see, e.g., Maggiolini et al., 2003; DeCicco, 2007; Bulkeley and Domhoff, 2010).

Many research findings seem to confirm the so-called “continuity hypothesis” (see, e.g., Domhoff, 1996; Kramer, 2006; Pesant and Zadra, 2006; Schredl, 2006; Skancke et al., 2014), which states that dreams closely reflect waking life. In fact, a number of connection patterns between waking life and dreaming have been described, regarding emotion, stress, level of well-being, personal characteristics, and psychopathology.

The just mentioned recent progresses in the scientific study of dreaming suggest that dreaming can be inserted without discontinuity into the mainstream of current neuroaesthetical research. In fact, on the physiological side, both dream research and neuroaesthetical research have applied powerful neuroimage methods, while on the psychological side the dream experience has been studied with quantitative methods similar to those applied to the waking experience. In the light of the continuity hypothesis, and in particular of the fact that perceptual modalities that dominate in wakefulness are also dominating in dreams, it can be reasonably assumed that basic aspects of dreaming can play a role in a number of waking experiences, specifically in esthetic experiences.

Very different modalities of consciousness can characterize wakefulness: typical well-known examples are day-dreaming and particular psychophysiological conditions such as meditation, as well as striking phenomena of dissociation between explicit and implicit memory. Different levels of consciousness characterize the dreaming state as well: the most remarkable example is given by lucid dreaming, i.e., sleep periods during which the dreamer is aware of actually dreaming (LaBerge, 2000). Furthermore, it has been suggested that dreaming in NREM sleep is related to “covert” REM processes that occur locally in the brain (Nielsen, 2000: Nir and Tononi, 2010). The phenomenon of dreaming appears therefore to offer interesting clues for the study of consciousness; this idea has been recently underlined from a philosophical point of view by Metzinger (2013).

In the next section, some indications are given for a possible neuroaesthetical analysis of art products founded on our knowledge of the dreaming phenomenon.

## Basic Features of Dreaming that can Give Effective Tools for the Neuroaesthetical Study of Artistic Products

Recent research has recognized and described basic features of dreams that can provide effective clues for the insertion of concepts proper for the study of dreams into the discipline of Neuroesthetics.

According to Hartmann (2008), a “central image” characterizes many dreams: this image “can often lead very quickly to an important underlying emotion or concern, which is often immediately recognizable, but sometimes is unexpected or even unconscious to the dreamer”. A typical example of central image that led the dreamer to represent it artistically is given by a watercolor by Dürer in the Kunsthistorisches Museum of Vienna. Below the watercolor Dürer wrote: “In 1525, during the night between Wednesday and Thursday after Whitsuntide, I had this vision in my sleep, and saw how many great waters fell from heaven. The first struck the ground about four miles away from me with such a terrible force, enormous noise and splashing that it drowned the entire countryside. I was so greatly shocked at this that I awoke before the cloudburst.”

A frequent phenomenon in dreams is that which Freud called “condensation” and defined in the following terms: “You will have no difficulty in recalling instances from your own dreams of different people being condensed into a single one. A composite figure of this kind may look like A perhaps, but may be dressed like B, may do something that we remember C doing, and at the same time we may know that he is D.” (Freud, 1973, p. 206; the lectures were delivered in 1915–1916 and 1916–1917). Cognitive and therapeutic studies of dream have confirmed the general existence of condensation phenomena in dreams: this data has a phenomenological value, which is independent from the validity of the theory in which Freud inserted his observations. In particular, Hartmann (1998, 2010) has underlined the frequent occurrence of condensation with examples taken from his own experience and from the literature and has interpreted this phenomenon theoretically as a manifestation, enhanced during dreaming, of the basic brain property of making connections. Chapter 7 of Hartmann (2010) has the significant title “Connection as combination; connection as condensation; connection as metaphor; the dream as picture-metaphor”. Lakoff (1997) has interestingly inserted condensation into the framework of cognitive psychology. The phenomenon of condensation directly regards aspects of the dreamer’s life: it is therefore closely connected to the continuity hypothesis.

Patterns of connections among different memory sources have been described (Barcaro et al., 2005; Barcaro, 2010): these connections, which can be identified by textual analysis of associations with the dream, often regard distant episodes of the dreamer’s life and often surprise the dreamer himself or herself. These patterns establish unexpected, often enlightening, correspondences between the different sources. A heuristic rule can be applied to account for the patterns of links among sources: they are such that the possible difficulties which the dreamer is meeting in his or her real life are somehow made less hard, and the positive aspects of the situation in which he or she stays are somehow underlined and made more important. The recognition of these link patterns often allows two interesting phenomena to be observed: the existence of a second, more important, present concern additional to that which the dreamer has first indicated (“shift of the present concern”), and the actual realization, in the dream experience, of the context changes implied by the link pattern.

Phenomena of self-similarity (a property exhibited in particular by fractals) have been observed in artistic products: for instance, Taylor et al. (1999) analyzed the fractal content of Pollock’s drip paintings, finding that the fractal dimensions increased steadily through the years. Self-similarity is a property of self-organizing systems and therefore its occurrence can shed light on significant brain mechanisms. According to a hypothesis advanced and formulated in mathematical detail by Kahn and Hobson (1993), abrupt shifts in neural activity patterns during ponto-geniculo-occipital (PGO) bursts of neural activity are related to discontinuous jumps between dream events. Barcaro and Rizzi (2010) observed self-similarity phenomena in dreams at three scale levels: (a) connections among different sources of a single dream; (b) connections among different source clusters of a single dream; (c) connections among different dreams of a single dreamer.

A number of the dream-like properties just mentioned can be recognized in visual works by focusing on their main Gestalt properties. Indeed, the study of dream-like properties in artistic products, far from being alternative to, is necessarily based on a previous analysis of perceptual features.

As we will see in the examples given in the next section, forms of interaction between different levels of consciousness with regard to dreaming have often characterized the creation of artistic products; in these cases, conscious references to dreaming have facilitated the unconscious insertion of dream-like features on the part of the artists and, consequently, the unconscious recognition of these features on the part of the observers.

Studies based on the idea of a connection between dreaming and artistic creation can take advantage of detailed historical documentation, regarding the purposes of the artists, the purposes of the commissioners, the artists’ experiences, and the cultural and social contexts. Indeed, a neuroaesthetical approach including the recognition of dream features can allow a more easily overcoming of the frequent dichotomy (discussed in particular by Bullot and Reber, 2013) between psychological and historical methods.

## Examples of Application of the Proposed Approach

We feel that the effectiveness of the method we are proposing can be best tested on works that present significant dream-like features although they do not represent dreams explicitly. The role of these features in artistic creation is generally mostly unconscious. We limit ourselves to considering four famous instances: Giorgione’s “Tempest”, Leonardo da Vinci’s “Virgin of the rocks”, Vermeer’s “Music lesson”, and Millais’ “Christ in the house of his parents”.

### Giorgione’s “Tempest”

Giorgione’s “Tempest” (storm), a masterpiece of the Venetian Renaissance realized at the beginning of the 16th century, is housed in the Gallerie dell’Accademia in Venice.^1^ Its name derives from a short description given in a catalog drafted in 1530: “small village on canvas, with the storm, the gypsy woman, and the soldier”. The painting includes: a sky darkened by an impending storm; a stream flowing slowly and tortuously from the distance to the foreground, or perhaps in the opposite direction; a level bridge, supported by low dark pillars; the palaces and the towers of a village or town, along the right (as seen by the observer) side of the stream; a white bird, perhaps a stork, on the top of one of these buildings; a naked young woman nursing a child in the foreground at the right, separated from the town by high trees and a bush; a ruined ancient wall with blind arches and a slightly inclined low wall supporting two broken pillars on the left side; a young man standing, lavishly dressed, holding a long staff, perhaps a lance, at the left, in the foreground. The painting appears as somehow illogical and certainly enigmatic. The literature about the “Tempest” includes a number of often interesting and well-argued proposals of interpretation, none of which has however so far achieved the status of being generally accepted. The considerations that we are developing do not depend on any particular interpretation.

A definite conclusion has been achieved about the village, or rather the town, which is certainly Padua (Guidoni, 1995).Kaplan (1986) interpreted the storm as a “martial metaphor” of the battle between the Venetians and the League of Cambrai, whose forces besieged Padua in September 1509: inside the metaphor of the storm, a lightning bolt in the dark sky can be interpreted as a metaphor of the fire of the besieging artillery. With regard to the monuments in the left part of the painting, the hypothesis appears very plausible (Paoli, 2011a) that the tall wall with blind arches represents the façade of the Palace of the “Porphyrogenitus” in Constantinople, and that the wall with two broken pillars represents the “Diplokionion” (double pillar), again in Constantinople. Paoli has indicated a precise historical event that accounts for the presence of monuments of Constantinople: slightly before the siege of Padua, in the same month, a terrible earthquake, followed by a tsunami, devastated Constantinople, killing more than ten thousand people and destroying houses and mosques. It happened, however, that all the Venetians that were there were unharmed, and none of their houses were ruined. In the painting, the tall wall is inclined because of the earthquake.

A basic dream-like character is given by the establishment of a connection between two episodes, i.e., the Constantinople earthquake and the siege of Padua. The link between the earthquake and the war is figuratively represented by the storm, in the same way as a central image often expresses the emotional content of a dream. According to the heuristic rule indicated above, negative contents in the memory sources of a dream are made less negative, if not completely reversed into positive, by the dream experience itself. If this criterion is applied to the dream represented in the “Tempest”, the following implication can be obtained: in the same way as, in the case of the earthquake, none of the Venetians who were in Constantinople were killed and none of their houses were destroyed, the war against the League of Cambrai will be successful for the Venetians. This explains why this painting, although being called “the Tempest”, does not represent any tempest: the storm is only imminent and has not yet broken. Indeed, according to the dream-like significance of the painting, no storm will break. The woman is calm despite the impending storm, as the young man is: both of them seem to have the inner knowledge that no danger actually exists.

Both the figure of the young woman and that of the young man offer a remarkable example of dream-like condensation. In fact, she has a determined human physiognomy, perhaps she is a gypsy; at the same time, the circumstances of her presence being undetermined, she generally represents a nursing mother. The young man could be the commissioner, or a soldier, or a Venetian noble, or generally a person reassured by a dream. The image of the nursing woman suggests that the heuristic rule can be immediately extended: the dreamer (or the Venetians, or anybody) will be saved from the current difficulties in the same way as they were saved by a nursing mother as children. This extension corresponds to the phenomenon, described in the previous section, called “shift of the present concern”: the dream-like significance of the painting therefore develops into a general, universal value. In particular, it is not limited to the gender specification of the dreamer as a male. In fact, in the same way as the earthquake and the war struck men and women equally, the figure of the nursing mother is obviously universal. For this reason, the considerations that we are developing also hold for the previous project of the composition, showed by X-ray analysis of the canvas, which included the image of a bathing woman, later replaced by the young man. As a result of these complex aspects of condensation, the painting appears to establish connections between events that are distant in space and in time, in the same way as dreams reveal connections between different episodes in the dreamer’s life.

Aspects of figurative self-similarity can be observed in the bushes and the trees behind the young woman. Moreover, an elementary but artistically powerful form of figurative self-similarity at different scales can be recognized by analyzing the condensation property of the stream. First, it separates the two parts of the composition, the young man and Constantinople being at the left, and Padua and the young woman being at the right. Second, its flowing from a distance suggests the flowing of time, the church recognizable as the Carmine Church of Padua being very far, and the human figures being close. Thirdly, its bending contributes to the atmosphere of calmness given by the painting, in spite of the impending storm.

The painter was mostly unaware of these dream-like features, which are among the reasons for the emotional reactions of the observers. However, historical research has strongly supported the hypothesis that Giorgione’s conscious purpose was that of representing a dream. Lettieri (1994) suggested that the theme of the “Tempest” was inspired by the passage of Sannazzaro’s pastoral romance “Arcadia” where Mother Earth appears in a dream to Sincero, the first character. Among the likely literary sources of Sincero’s dream, Apuleius’ Latin novel “Metamorphoses” (also called “The golden ass”), written in the 2nd Century AD, is particularly important. In Book XI, Lucius, the first character of the novel, who by mistake has turned himself into an ass, has the vision of Isis during a dream. The goddess, whom Lucius invokes as the synthesis of all the goddesses representing Mother Earth, tells him how to return to human form by participating in a religious procession, called “Isis’ vessel”, the following day. According to Paoli (2011a), the nursing woman represents directly Isis and indirectly Mother Earth. The cultural atmosphere of the time accounts for the “Tempest” being inspired by a literary dream. In fact, a diffuse interest in dreaming characterized the Venetian, and more generally Italian, society in the 16th Century (for a review, see Paoli, 2011b). A complex relationship existed in the mind of Giorgione between conscious and unconscious processes: the cultural context attributed basic value to dreams; dream representation was very likely the basic conscious purpose of his work; this context and this purpose exerted a successful facilitation, a form of priming, on the unconscious establishment of precise similarity elements between his painting and a dream.

In the light of the connection of the “Tempest” with Sincero’s and Lucius’ dreams, the condensation value of the nursing mother is extended: she is a religious entity, a goddess, Isis or Mother Earth. If, on the basis of the heuristic rule, we assume that dreams often express a wish for forms of salvation, we can say that dreams can present a latent or overt “spiritual” aspect, where the word “spiritual” has no metaphysical implications, only indicating an idea of salvation connected to the deep feelings of the dreamer. Goguen (1999) supported the idea of a “spiritual perspective”, perfectly compatible with a neuro-scientific approach, in the study of art. Indeed, important studies about dreaming have focused on their spiritual content (Bulkeley, 2008, 2009) and on their value in the lives of the dreamers (Siegel, 2002).

### Leonardo da Vinci’s “Virgin of the Rocks”

Leonardo’s “Virgin of the rocks”, housed in the Louvre Museum,^2^ was probably painted in the years 1483–1486 (Kemp, 1981) after having been commissioned by the “Confraternity of the Immaculate Conception” in Milan. A second version, which was very likely later, held at the National Gallery in London, presents differences in composition, color and shapes; in particular, the typical Leonardo’s “sfumato” technique, which consists in subtly blurring borders as smoke (Italian: “fumo”) does, is reduced in the copy at the National Gallery. We now only consider the Louvre version. In this painting, Leonardo did not directly refer to the doctrine of the Immaculate Conception, i.e., of Mary not sharing the humanity’s state of “original sin” derived from Adam’s rebellion in Eden. On the contrary, Leonardo referred to Jesus’ flight into Egypt to escape from the massacre of the innocents: according to a diffused narrative, John the Baptist escaped as well, escorted by an angel, and during his journey met Jesus. The group of four characters (Mary, Jesus, John, and the angel) is arranged into a pyramidal composition, Mary being at the apex. They are placed in a cave formed by rocks, and a distant landscape of mountains and waters appears in the background.

A major figurative property of the painting is given by three levels of reassuring enclosures. First, the group has found shelter in the inside of a cave. Second, the disposition of the characters and of the light, which falls on the four faces and on the bodies of the two children, creates an approximate circle; in fact, the basic pyramid formed (from the left) by John, Mary and the angel, is corrected into a bended line, thus suggesting a circle, by the figure of Jesus. Mary’s right arm greatly contributes to this second circle, by extending to reach John’s shoulder with her protecting hand. These two enclosures contain a third one: Mary’s mantle opens to reveal the luminous surface of the lining: this central image approximately corresponds to Mary’s womb. In this way, Leonardo offers a three-scale representation of motherhood, an artistic version of a matrioska toy. The innovative idea of inserting the group in a cave realizes a shift of the idea of salvation: not only are they escaping from Herod, but also they find protection inside a natural reassuring environment. In this way the idea of salvation, which is connected to the basic subject of the painting, i.e., the flight into Egypt, is greatly extended, thus assuming a universal value: this salvation is given by motherhood. Certainly, the cave can be seen as a symbol of maternal womb in its own right (Hartt, 1952); however, the artistic effect is obtained by means of figurative means, particularly by the Gestalt value of the disposition and illumination of the characters.

We thus find that the basic idea of motherhood is common to the seemingly mundane painting of Giorgione and to the seemingly institutional painting of Leonardo. Mary’s role in Leonardo’s painting is different from the doctrinal role connected to the Immaculate Conception: this role is changed into a natural role, that of generation. The detailed representation or geological and botanical forms completely realizes this basic shift: the images of plants emphasize their “pattern of growth, implying that the source of change is within matter and not transcendent of it” (Garrard, 1992).

A striking phenomenon of condensation of opposite elements (precisely: masculine/feminine) is given by the angel: according to the doctrine, angels have no sex; figuratively, the sexual ambiguity of the angel is accompanied by the representation of an ideal form of beauty. This condensation of masculine and feminine extends to the cave, whose irregular forms often present evident phallic shapes together with receptacles and openings. The painting presents unexpected forms of correspondence between different elements: the shape of the hair of the characters resembles that of the plants (indeed, both belong to nature), the bodies of the two children seem to mirror each other, the face of Mary is similar to that of the angel, the hands and fingers of the characters make gestures that exchange messages, whose meaning seems not to be completely understandable outside the sacred group. Other correspondences link the landscape to elements of the cave: the distant mountains seem to mirror the rocks of the cave; the texture of the water outside the cave figuratively connects this external liquid element to the mantle of Mary.

The protective role of the cave for Jesus and John assumes a further significance if we consider Leonardo’s biographical data: in fact, in addition to many other activities, he was a speleologist: he reports that once, in front of the entry of a cave, he had two contrasting feelings: “fear and desire to see if inside there was anything miraculous” (Marinoni, 1952; Emison, 1993). Certainly, the dream-like properties of the painting correspond to the original personal feelings of the artist: the fear of the escaping children corresponds to his fear, and the desire for something miraculous is realized by the salvation role of both the cave and Mary as symbols of motherhood.

In the same way as the dream-like features of the “Tempest” were facilitated by a conscious purpose of the painter connected to literary dreams, the original literary source of the Virgin of the Rocks was closely connected to a dream: according to the Gospel of Matthew, the flight into Egypt was due to a dream: an angel appeared to Joseph and told him to take the young child and his mother, and flee into Egypt. Certainly, Leonardo credited dreams (or dream-like fantasies) with great importance: he attributed his own “destiny” of being interested in the flight of birds to the infantile fantasy of a kite; Leonardo’s reminiscence of this childhood episode led Freud to a famous, although far from rigorous, interpretation of the painting “Virgin and Child with St. Anne”. A note in the Atlantic code is precisely “about dreaming”; furthermore, Leonardo’s “allegory with solar mirror” in the Louvre contains imaginary animals that can be viewed as intentionally representing bizarre dream images.

### Vermeer’s “The Music Lesson”

In Vermeer’s painting “The music lesson”, part of the Royal Collection at St. James’s Palace in London,^3^ a young woman, seen from the back, is playing the virginal (small harpsichord) and a man is listening to the music and observing the player. They are in a room filled of light coming from the left through the windows. Besides the two characters, the composition includes the virginal against the front wall, a marble floor, a table wholly covered by a Persian carpet at the right, a gilded plate and a white jar on the table, a chair with conspicuous tacks near the table, a bass violin lying on the floor, a painting on the right wall, and a mirror on the front wall; all of these elements are painted in detail, giving an idea of harmony and decorative richness.

An immediately perceivable property of the painting is its ambiguity: the man “could be her teacher, or brother, or husband or a suitor. They could be discussing something quite banal, like the quality of her playing or something a great deal more serious, such as a separation or a reconciliation. All these interpretations have equal force and validity” (Zeki, 2004). Certainly, this ambiguity is less connected to perceptively ambiguous images, such as the Kanizsa cube, than to the condensation proper of dreams. A subtler ambiguity regards the young woman: she seems concentrated on the keyboard, but her eyes, reflected from the mirror, appear to be looking at the man, possibly because she is checking if he approves, or possibly because of a feeling of affection (for a discussion about this point, see Kandel, 2012). The correspondence between the covert gaze of the woman and the man’s gaze, overtly directed to her, is in agreement with the dream function, expressed by the above formulated heuristic rule, of actually overcoming a negative, limited condition, given in this case by a relationship confined to teacher-pupil terms.

Unexpected dream-like properties of the “Music lesson” can be recognized if aspects of self-similarity are observed. A basic element of self-similarity at different scales is given by the insertion of a painting in the painting. In fact, the image on the wall, which is faded and only partially visible, and thus, like a dream, simultaneously conceals and reveals, is one of the Dutch works that were inspired by Rubens’ “Roman Charity”. At first sight, this is in agreement with the idea of the ethical educational purposes of teaching young ladies to play virginals. However, this meaning markedly changes if the specific episode of charity is considered: a woman, Pero, secretly breastfeeds her father, Cimon, incarcerated and sentenced to death by starvation. A correspondence is thus established between two couples: that of the playing woman and the man, and that of Cimon and his daughter. This correspondence can be understood in the light of the heurist rule: the man is metaphorically in chains, like Cimon, probably because his feelings must be kept secret or are anyhow constrained, but, in spite of this condition, his love desire can however be realized. The young lady’s charity consists in returning his feelings. Here, we find again a form of salvation, because Pero saved her father, although in the context of the painting the idea of salvation is slightly ironic, very mundane.

A number of correspondences between elements of the painting exist. In addition to the virginal, another instrument is figuratively important, the unattended bass viol on the floor before the couple, which seems to be waiting for somebody to play it. The textures of the carpet, of the veins of the marble, and of the decorations of the virginal correspond to each other, somehow resembling abstract paintings of the 20th century (Wheelock, 1981). In turn, the abstract aspect of these elements of the composition evokes the abstract character of music; indeed, an idea of music filling the room pervades the painting. Thus, the visual experience of observing the painting raises a kind of hallucinatory acoustic sensation, together with a tactile sensation conveyed by the texture of the decorated surfaces. In this way, the above mentioned neuroesthetical principle of “grouping” (Ramachandran and Hirstein, 1999) is extended to encompass a variety of sensory modalities. The carpet prevents the observer from seeing the table, which is concealed in the same way as part of the painting is and the eyes of the woman would be if there was no mirror.

The white pitcher plays an important visual role for both the absence of decorative texture and for its standing out color. Thus, although being placed far from the center of the scene, it unexpectedly plays the role of the central image of a dream. Indeed, the pitcher somehow synthesizes the dream-like polyvalence of the painting, being at the same time important and mysterious, because the observer will never know what its content is. Also in this case, it should be underlined that the dream-like features derive from basic perceptual features, because the figurative impact of the pitcher can be attributed to the above mentioned phenomenon of “peak shift”.

The characteristics of the “Music lesson” that we have observed are in agreement with the artist’s purpose, very direct in other paintings, of representing day-dreaming. This is particularly clear in the “Woman standing at a virginal” in the National Gallery in London: in that painting, the young lady, close to an empty chair, idly improvising at the virginal, with a large picture of Cupidus hanging on the wall, appears to be dreaming, or better day-dreaming, of her absent lover.

### Millais’ “Christ in the House of his Parents”

Millais’ “Christ in the house of his parents”, now housed in the Tate Gallery in London,^4^ was exhibited at the Royal Academy in 1850. It represents the Holy Family in Joseph’s carpenter’s shop. Jesus, a boy dressed in a white robe, has been injured by a nail: his left hand has been wounded and a drop of blood has fallen on his foot. Mary, his mother, kneeled beside him, offers him her cheek to kiss. Joseph, who has been working at a door placed on a large rectangular worktable, is examining the injured hand: the veins of Joseph’s arms protrude, his fingernails are dirty. There are other three characters: Anne, Jesus’ grandmother, who is reaching for pliers on the table; John the Baptist, who is carrying a bowl of water to wash the wound; and Joseph’s assistant. The floor is strewn with whorled wood shavings. A ladder, on which a dove is perching, is placed against the wall, where some carpenter’s tools are hanging, including a triangular set-square. In the background, a flock of sheep outside the shop are visible.

Each element has a clear symbolic value: the water in the bowl stands for the baptism, the flock for the Christian community, the assistant for the apostles, the dove for the Holy Spirit, and the set-square for the Trinity. But the main references, immediately understandable, regard the future Passion of Jesus. This symbolism was certainly conscious on the part of Millais. However, the condensation value of the various elements of the painting is much more complex. Indeed, what is represented in the picture is not merely a coherent set of symbols, but is also a realistic picture of what might likely have happened, and perhaps did happen, during Jesus’ childhood. This creates a feeling of mystery and miracle, because symbolism and realistic representation for some reasons coincide.

The condensation value of the various items thus establishes a dream-like correspondence between two episodes in Jesus’ life: in fact, being wounded in his father’s shop provides an anticipation of his sufferance on the cross. Applying the above stated heuristic rule, we find that a basic dream-like element of the scene is Christ’s serenity, in spite of the wound: in this way, the painting assumes a spiritual significance, expressing the idea, proper of the Christian doctrine, that Christ voluntarily sacrificed himself to save humanity. The serenity of the child appears, however, as directly due to the tender attitude of his parents, which conveys a feeling of eternal parental love.

The polyvalence of the carpenter’s shop is not only due to its being Joseph’s shop and being a symbolic representation of the Golgotha as well, because it also represents a typical carpenter’s shop in England in the 19th Century. In this way, the condensation value of the composition is threefold, and a remarkable form of shift of the implied change of context takes place, establishing a precise correspondence between the conditions of the Holy Family and those of humble people.

This painting, together with other Pre-Raphaelites paintings exhibited in 1850, arose a violent reaction on the part of the establishment; “the critics suddenly abandoned their measured tones to engage in what can only be called a campaign of vituperation” (Prettejohn, 2000). On May 9, 1850, the critic of “The Times” wrote: “The attempt to associate the Holy Family with the meanest details of a carpenter’s shop, with no conceivable omission of misery, dirt, or even disease, all finished with the same loathsome minuteness, is disgusting.” Also Dickens reacted violently to Millais’ painting. Indeed, “the net result of Millais’ treatment was to flaunt the conventional idea of the Holy Family and violate the Victorian code of propriety” (Boime, 1975). In the same way as a dream can contain “disgusting”, “revolting” items (using the same terms of the 1850 critics), Maillais’ painting represented “loathsome minuteness”: in the dream-like perspective, and from the point of view of the observers of today, this minuteness acquires artistic value.

A further application of the heuristic rule can be performed considering the flock of sheep. Certainly, they are not in the sacred place reserved to the Holy Family, in the same way as the lives of ordinary people are definitely constrained. But this limitation is completely overcome by the fact that the living and working place of the Holy Family shares the humble conditions of ordinary people.

Finally, we can consider the condensation value of the ladder. Similarly to the other items of the composition, it presents three meanings: it is a ladder in Joseph’s shop, it is the ladder which helped to depose the body of Christ from the Cross, and it is a ladder in an 18th-century carpenter’s shop. But it has a fourth significance, of which the painter was very likely conscious: it evokes Jacob’s dream in the Genesis: he saw angels going up and down a ladder between heaven and earth. In other words, also in this case we find that different levels of consciousness with regard to the dreaming phenomenon co-existed in the mind of the artist and influenced each other.

## Short Conclusions

The purpose of this study has been to support the idea that an insertion of issues connected to the dream experience can be useful for the advancements of Neuroesthetics. A historical perspective can lead to a better understanding of the reasons why the poor role so far played by dreaming in Neuroesthetics, far from representing a limitation, has proved scientifically fruitful: basically, this has allowed the researchers to concentrate on fully exploiting great scientific advancements achieved in the study of perception. In the same perspective, it appears that the conditions that have determined the poor role of dreaming can now be overcome. Certainly, Neuroesthetics is a recent discipline and is open to the introduction of many new approaches and viewpoints; however, we feel that the issue of dreaming should be credited with a privileged significance for the future developments of this discipline, in the light of the basic function implicitly or overtly fulfilled by dreams in artistic production and by the creative aspects of dreams often underlined by dream scholars.

An approach to the study of visual artistic products is proposed consisting in the recognition of well-defined features which have been assessed by scientific research as significant for the dream experience. A further criterion for the selection of these features has been that they should be connected to basic properties of visual perception, thus making them appropriate for the study of visual art. This latter property highlights that an insertion of dreaming into Neuroesthetics can be made without discontinuities in the mainstream of this discipline, which has been characterized by a fundamental role played by analyses of perception modalities.

An application of the proposed method to four recognized masterpieces seems to indicate that this approach can be fruitful; we have observed that aspects proper of the dream phenomenon have been not only present, but also important, in the creative process, and have been active at different, and interacting, consciousness levels in the minds of the artists.

## Conflict of Interest Statement

The authors declare that the research was conducted in the absence of any commercial or financial relationships that could be construed as a potential conflict of interest.

## Footnotes

^1^www.gallerieaccademia.org

^2^www.louvre.fr

^3^www.royalcollection.org.uk

^4^www.tate.org.uk
